# Association of maternal dietary cholesterol intake during the second and third trimesters of pregnancy and blood glucose and pregnancy outcome in women with gestational diabetes mellitus: a prospective cohort study

**DOI:** 10.3389/fnut.2024.1449000

**Published:** 2024-12-12

**Authors:** Cuiling Xie, QingXiang Zheng, Xiumin Jiang, Yanping Liao, Xiaoxia Gao, Yu Zhu, Jianing Li, Rulin Liu

**Affiliations:** ^1^School of Nursing, Fujian Medical University, Fuzhou, China; ^2^Nursing Department, Fujian Maternity and Child Health Hospital College of Clinical Medicine for Obstetrics & Gynecology and Pediatrics, Fujian Medical University, Fuzhou, China; ^3^Nursing Department, Fujian Obstetrics and Gynecology Hospital, Fuzhou, China; ^4^Pediatric Intensive Care Unit, Guangzhou Women and Children's Medical Center, Guangzhou Medical University, Guangzhou, China

**Keywords:** gestational diabetes mellitus, cholesterol, dietary, blood glucose, pregnancy outcome

## Abstract

**Background:**

Cholesterol is essential for pregnant women to maintain maternal health and fetal support development. This study aimed to assess the cholesterol intake of women with gestational diabetes mellitus (GDM) during the second and third trimesters of pregnancy and to explore its effects on blood glucose and pregnancy outcomes.

**Methods:**

This prospective cohort study collected dietary data using a food frequency questionnaire (FFQ) administered during the 24–30 gestational weeks (first survey) and the 34–42 gestational weeks (second survey). Blood glucose parameters and pregnancy outcomes were obtained from electronic medical records. Participants were divided into two groups according to the median cholesterol intake: low and high cholesterol intake groups.

**Results:**

GDM women generally consumed high levels of cholesterol during pregnancy, with intake increasing in the third trimester compared to the second trimester. Compared to women with high cholesterol intake, GDM women with low cholesterol intake had a higher risk of abnormal hemoglobin A1C (HbA1C) during the second trimester [OR 26.014 (95% CI 2.616–258.727)] and the third trimester [OR 2.773 (95% CI 1.028–7.482)], as well as abnormal fasting blood glucose during the third trimester [OR 2.907 (95% CI 1.011–8.360)]. Furthermore, in the second trimester, GDM women with high cholesterol intake had higher risks of macrosomia [OR 23.195 (95% CI 2.650–203.024)] and large for gestational age (LGA) [OR 3.253 (95% CI 1.062–9.965)] but lower risks of small for gestational age (SGA) [OR 0.271 (95% CI 0.074–0.986)] compared to those with low cholesterol intake. However, in the third trimester, GDM women with high cholesterol intake had lower risks of macrosomia [OR 0.023 (95% CI 0.001–0.436)] and LGA [OR 0.199 (95% CI 0.042–0.949)].

**Conclusion:**

Cholesterol intake among GDM women during pregnancy was associated with blood glucose control and significantly influenced the risks of macrosomia, LGA, and SGA. However, LGA and SGA were also influenced by pre-pregnancy BMI, indicating cholesterol intake was one of multiple contributing factors. Limiting cholesterol intake may help GDM women better manage blood glucose levels and mitigate adverse pregnancy outcomes.

## 1 Introduction

With the global epidemic of obesity and metabolic disorders, the incidence of gestational diabetes mellitus (GDM) has been increasing for decades (1), making GDM the most common complication during pregnancy (2, 3). GDM refers to any degree of glucose intolerance that occurs or is first discovered during pregnancy (4). According to the 10th edition of the International Diabetes Federation, 21.1 million women (16.7%) who gave birth to live babies worldwide in 2021 suffered from some form of hyperglycemia in pregnancy (HIP), with 80.3% of these cases (16.98 million) attributed to GDM (5). A 2019 systematic review and meta-analysis estimated the overall incidence of GDM among women in mainland China at 14.8%, based on the International Association of Diabetes and Pregnancy Research Groups criteria (6). In addition, GDM has been associated with higher risks of large for gestational age (LGA), macrosomia, and premature delivery (7). The risk of macrosomia in GDM women is nearly four times higher than in women with normal pregnancies, with an incidence of macrosomia in GDM women reported at 25–40% (8). Furthermore, women with GDM face a 10-fold increased lifetime risk of developing type 2 diabetes (9).

Cholesterol plays a vital role in intracellular material transport, cell signaling, and nerve signal transduction (10, 11). Cholesterol is also involved in many other important physiological processes. For example, cholesterol is the precursor of steroid hormones, bile acids and vitamin D in human body (12, 13). Therefore, appropriate cholesterol levels are particularly important for maintaining body health. The small intestine is the primary site of cholesterol absorption in the human body. The main sources of cholesterol are taken from the diet, which mainly includes animal foods such as eggs, beef, pork, poultry, fish and shrimp, dairy products, and animal offal (14). Several metabolic studies have verified that dietary cholesterol does increase serum cholesterol concentration (15, 16).

Maternal cholesterol is important for fetal membrane function and fetal nervous and aortic system development (17, 18). Maternal total cholesterol level is considered one predictor of fetal dysplasia (19, 20). In traditional Chinese dietary culture, women are encouraged to eat more eggs, meat, and milk during pregnancy to ensure the intake of high-quality proteins and micronutrients. These foods are also a good origin of cholesterol-rich foods. This has led to a significant increase in dietary cholesterol intake during pregnancy, potentially elevating serum cholesterol concentrations (15, 16). Castro et al. found that pregnant women with high cholesterol intake were more likely to give birth to LGA newborns than women with low cholesterol intake (21). Wu et al. showed that an increase in total cholesterol in the maternal diet increased fasting blood glucose and 1-h postprandial blood glucose (22). However, there was no related research on the relationship between dietary cholesterol intake during pregnancy and blood glucose control or pregnancy outcomes in GDM women. Therefore, this study aimed to investigate the cholesterol intake of GDM women during the second and third trimesters of pregnancy and to explore its effects on blood glucose and pregnancy outcomes during the second and third trimesters of pregnancy to provide a reference for the formulation of dietary cholesterol recommended intake for GDM women in the future.

## 2 Materials and methods

### 2.1 Study design and participants

This study was designed as a prospective cohort study. It was conducted at the Fujian Maternal and Child Health Hospital and Fujian Obstetrics and Gynecology Hospital from 2 January 2022 to 30 December 2022. The convenient sampling method was used to select pregnant women who were diagnosed with GDM in the hospital. The diagnostic criteria for GDM were as follows (23): For women who were not previously diagnosed with diabetes, a 75 g oral glucose tolerance test (OGTT) was performed at 24–28 weeks of pregnancy. Plasma glucose levels were measured on an empty stomach, as well as 1 h and 2 h after drinking sugar water. The plasma glucose thresholds were 92 mg/dL (5.1 mmol/L), 180 mg/dL (10.0 mmol/L), and 153 mg/dL (8.5 mmol/L) at fasting blood glucose, 1 h and 2 h blood glucose after drinking sugar water, respectively. When any of the three test results reached or exceeded the plasma glucose threshold, the diagnosis of GDM was made. The exposure in this study was high cholesterol intake in the second and third trimesters of pregnancy. The control group was women with GDM with low cholesterol intake.

The inclusion criteria for participants were as follows: (1) women were diagnosed with GDM, the diagnostic criteria according to the American Diabetes Association issued by the “Standards of Medical Care in Diabetes-2022” (23); (2) aged between 20–45 years old, no disturbance of consciousness, who could fill in the questionnaire; (3) routine prenatal examination and planned to give birth in the unit of this study; (4) they volunteered to participate in this study. Meanwhile, the exclusion criteria were also as follows: (1) GDM women with severe pregnancy complications or complications, such as pregnancy with heart disease; (2) women suffering from heart, liver, kidney, and other important organ diseases; (3) GDM women who were currently on antidiabetic medications or insulin therapy. Additionally, the shedding criteria were as follows: patients with incomplete gestational blood glucose data and pregnancy outcome data.

The treatment of GDM women in this study was diet management. Obstetricians and nutrition doctors would carry out personalized diet management based on the examination results of their body indicators and diet intake situation. All participants completed the first questionnaire at 24–30 weeks of gestation, and then the researchers followed up until 34–42 weeks of gestation to conduct a second questionnaire survey. The questionnaire included general information and a food frequency questionnaire (FFQ). General information includes social demographic data, reproductive history, disease history, family history, diet and living habits, etc. Among them, pre-pregnancy height and weight are self-reported pre-pregnancy height and weight of pregnant women during the first prenatal examination. The height and weight values were re-measured and corrected by trained professional staff to ensure the authenticity and reliability of the data. They were extracted from participants’ medical charts. Body mass index (BMI) was expressed as a weight ratio in kilograms to the square of height in meters (kg/m^2^). The FFQ used in this study was designed according to the dietary habits of the Chinese people (24). It included 13 categories of foods (including staple foods, beans, vegetables, algae, fruits, dairy, meat, aquatic products, eggs, snacks, beverages, and cooking oils and condiments), a total of 70 food items, involving more than 200 common foods, which could well reflect the dietary status of the study participants (24). The researchers asked the subjects about their dietary intake in the past 3 months of the second trimester and the past 3 months of the third trimester through face-to-face interviews. The FFQ collected in the first survey reflected the dietary intake of women with GDM before diet management in the second trimester of pregnancy, while the FFQ collected in the second survey reflected the dietary intake of women with GDM after diet management in the third trimester of pregnancy.

All investigators were trained by nutrition experts on how to use FFQ. All investigators were required to be proficient in guiding interpretation terms, data collection content, and inquiry skills, and they adopted consistent investigation order and inquiry methods in the investigation process to ensure the reliability and consistency of the survey data. The researchers used three-dimensional food models and a ‘Retrospective dietary survey auxiliary reference recipes’ to help pregnant women accurately recall the dietary intake of the second and third trimesters of pregnancy.

The survey was conducted following the Strengthening the Reporting of Observational Studies in Epidemiology (STROBE) statement.

### 2.2 Blood glucose index

Blood glucose indicators were obtained by querying electronic medical records. Blood glucose indicators include blood glucose during the second and third trimesters of pregnancy. For the second trimester of pregnancy, the blood glucose indicators included fasting blood glucose, 1 h and 2 h of blood glucose after drinking sugar water, and glycosylated hemoglobin A1C (HbA1C) levels, which were all detected at 24–28 weeks of pregnancy.

For the third trimester of pregnancy, the blood glucose indicators included venous fasting blood glucose, venous postprandial 2-h blood glucose, and HbA1C levels, which were detected when the pregnant woman was admitted to the hospital for delivery (36–40 weeks). The target of venous fasting blood glucose control was <5.3 mmol/L, the target of 2-h postprandial blood glucose control was <6.7 mmol/L, and the target of HbA1C level control was <6% (25).

### 2.3 Pregnancy outcomes

Pregnancy outcomes were obtained by querying electronic medical records. Pregnancy outcomes included neonatal birth weight, macrosomia, SGA, and LGA. Newborns weighing more than 4,000 g are macrosomic (26). Neonates with birth weight below the 10th percentile of the average weight of the same sex and the same gestational age are SGA (27), while neonates above the 90th percentile are LGA (28).

### 2.4 Ethical considerations

This study has been approved by the Ethics Committee on research hospital (Approval No.2021KR041 and No.2021KLRD645). Researchers were responsible for making respondents fully understand the purpose, methods, and possible harm of this study. Respondents volunteered to participate in this study and had the right to informed consent. Additionally, all participants provided written informed consent.

### 2.5 Statistical analysis

In this study, Epi Data 3.1 was used for data entry, and then researchers manually analyzed it in Excel. The average daily intake of each food = (eating frequency × single eating weight) / eating interval days. To improve the accuracy of calculated intakes for cholesterol, total energy, saturated fatty acids, monounsaturated fatty acids, and polyunsaturated fatty acids, investigators first evaluated the dietary intake of GDM participants using the FFQ. Then, they calculated the contents of cholesterol, total energy, saturated fatty acids, monounsaturated fatty acids, and polyunsaturated fatty acids in various foods according to the ‘China Food Composition Table Standard Edition’ (29). Finally, the total cholesterol intake, total energy, saturated fatty acids, monounsaturated fatty acids, and polyunsaturated fatty acids in GDM women during the second and third trimesters of pregnancy were obtained. Energy-adjusted daily intakes of cholesterol, total energy, saturated fatty acids, monounsaturated fatty acids, and polyunsaturated fatty acids were obtained using the residual method (30). The above methods refer to the research of Gong et al. (31).

There are no relevant guidelines or books, including *Dietary Reference Intakes for China* (2023), that recommend or limit cholesterol intake for pregnant women during pregnancy (32). Xue et al. investigated the dietary cholesterol intake of 4,146 pregnant women, grouping participants based on tertiles of total dietary cholesterol intake (33). However, due to the relatively small sample size of women with GDM in our study, we aimed to better capture cholesterol intake exposure in this population during both the second and third trimesters of pregnancy. Therefore, participants in this study were divided into two groups according to the median of cholesterol intake: the low cholesterol intake group and the high cholesterol intake group.

We compared the high cholesterol intake group with the low cholesterol intake group (the control group) to explore the effects of cholesterol intake level on blood glucose and pregnancy outcome in GDM women during the second and third trimesters of pregnancy. Age, nationality, pre-pregnancy BMI, number of pregnancies, number of deliveries, smoking history, education level, whether to work during pregnancy, family *per capita* monthly income, exercise, diet, sleep and living conditions, family history of obesity, family history of diabetes, family history of hypertension, GDM before pregnancy, history of hypertension, abnormal lipid metabolism, pre-pregnancy presence of thalassemia, thyroid metabolic disease, OGTT test results, neonatal gender, total energy, protein, carbohydrate, saturated fatty acid, monounsaturated fatty acid, polyunsaturated fatty acid intake were used as potential confounding factors for mid-pregnancy data analysis. The data analysis in the third trimester corrected the blood glucose control mode based on the above model. Among them, age and total energy, saturated fatty acids, monounsaturated fatty acids, and polyunsaturated fatty acid intake were used as continuous variables. The odds ratios (ORs) and 95% confidence intervals (CIs) were estimated for independent variables of regression analyses. And the *p* value of 0.05 or less was considered statistically significant.

## 3 Results

### 3.1 Characteristics of participants

A total of 400 Chinese women with GDM were included in this prospective study and completed the questionnaire survey. However, 42 cases were excluded because they went to another hospital to give birth, their pregnancy outcomes could not be obtained, or their data were incomplete. A total of nine cases declined to provide follow-up information on their pregnancy outcomes. A total of 349 women with GDM completed the first survey and were included to analyze in the second trimester of pregnancy. Among the 349 participants in the second trimester of pregnancy, 54 pregnant women refused to accept or had no time to accept the second survey in the third trimester of pregnancy, so we could not obtain their dietary data in the third trimester of pregnancy, and then they were excluded. Finally, 295 women with GDM completed the survey and were included in the third-trimester analysis.

The critical value of low and high cholesterol intake in the second trimester of pregnancy was 651.22 mg/d, and the critical value of low and high cholesterol intake in the third trimester of pregnancy was 673.28 mg/d. The characteristics of the participants are shown in Table 1. The average age of GDM women was 31.74 years old. Most of them lived with their husbands (77.94%) and had a job (75.07%) during pregnancy. About half of them had a bachelor’s degree or above (51.58%). In total, 69.34% of GDM women had a monthly income of more than 6,000 yuan (1 yuan = 0.1446 USD), and 58.07% of them had a normal BMI. The primipara accounted for 61.60%, and the cesarean section accounted for 15.47%. In addition, participants only differed in BMI, while there were no differences in other demographics, personal health status, family history, and pregnancy habits (Table 1).

**Table 1:** The characteristics of 349 GDM women were analyzed according to the level of cholesterol intake in the second trimester of pregnancy.

Characteristics	Total cholesterol intake		*Z*/χ^2^	*p*
	Less intake	More intake		
	*n* = 175	*n* = 174		
Baseline characteristics				
Age	31.92 ± 3.64	31.56 ± 4.18	−0.911	0.362
Nation			0.737	0.391
Han nationality	170 (97.1)	166 (95.4)		
Minority	5 (2.9)	8 (4.6)		
Education level			3.078	0.380
Junior high school and below	15 (8.6)	17 (9.8)		
High school or technical secondary school	26 (14.9)	18 (10.3)		
College for professional training	41 (23.4)	52 (29.9)		
Bachelor’s degree and above	93 (53.1)	87 (50)		
Work during pregnancy			1.310	0.252
No	39 (22.3)	48 (27.6)		
Yes	136 (77.7)	126 (72.4)		
Family *per capita* monthly income			0.353	0.838
<6000yuan	55 (31.4)	52 (29.9)		
6,000 ~ 8999yuan	63 (36.0)	68 (39.1)		
≥9000yuan	57 (32.6)	54 (31.0)		
Couples living together			0.025	0.875
No	38 (21.7)	39 (22.4)		
Yes	137 (78.3)	135 (77.6)		
Body mass index (kg/m^2^)			9.966	**0.007**
<18.5	20 (11.4)	20 (11.4)		
18.5–23.9	115 (65.7)	88 (50.6)		
≥24	40 (22.9)	66 (37.9)		
Number of pregnancies			0.024	0.878
First pregnancy	73 (41.7)	74 (42.5)		
Multiple pregnancies	102 (58.3)	100 (57.5)		
Primiparous woman			0.382	0.536
Yes	105 (60)	110 (63.2)		
No	70 (40)	64 (36.8)		
Cesarean section experience			0.830	0.362
No	151 (86.3)	144 (82.8)		
Yes	24 (13.7)	30 (17.2)		
Smoking history			/	0.499 ^*^
No	173 (98.9)	174 (100.0)		
Yes	2 (1.1)	0 (0.0)		
Family history of obesity			2.034	0.154
No	161 (92.0)	152 (87.4)		
Yes	14 (8.0)	22 (12.6)		
Family history of diabetes			0.784	0.376
No	116 (66.3)	123 (70.7)		
Yes	59 (33.7)	51 (29.3)		
Family history of hypertension			0.008	0.929
No	128 (73.1)	128 (73.6)		
Yes	47 (26.9)	46 (26.4)		
Pre-pregnancy history of gestational diabetes mellitus			0.357	0.550
No	162 (92.6)	158 (90.8)		
Yes	13 (7.4)	16 (9.2)		
History of pre-pregnancy hypertension			/	0.123 ^*^
No	175 (100.0)	171 (98.3)		
Yes	0 (0.0)	3 (1.7)		
Abnormal liver and kidney function before pregnancy			/	1.000 ^*^
No	172 (98.3)	172 (98.9)		
Yes	3 (1.7)	2 (1.1)		
Abnormal lipid metabolism before pregnancy			/	0.371 ^*^
No	174 (99.4)	171 (98.3)		
Yes	1 (0.6)	3 (1.7)		
Pre-pregnancy presence of thalassemia			1.968	0.161
No	166 (94.9)	170 (97.7)		
Yes	9 (5.1)	4 (2.3)		
Patients with thyroid metabolic diseases			0.873	0.350
No	167 (95.4)	162 (93.1)		
Yes	8 (4.6)	12 (6.9)		
Exercise frequency during the second trimester of pregnancy			0.576	0.902
None	45 (25.7)	44 (25.3)		
1 ~ 2 days/week	72 (41.1)	66 (37.9)		
3 ~ 5 days/week	43 (24.6)	48 (27.6)		
6 ~ 7 days/week	15 (8.6)	16 (9.2)		
The average sleep time in the past 3 months			1.758	0.185
<8 h	111 (63.4)	122 (70.1)		
≥8 h	64 (36.6)	52 (29.9)		
Extra meals during the second trimester of pregnancy			4.193	0.241
None	27 (15.4)	31 (17.8)		
1 ~ 2 days/week	64 (36.6)	72 (41.4)		
3 ~ 5 days/week	53 (30.3)	53 (30.5)		
6 ~ 7 days/week	31 (17.7)	18 (10.3)		
Daily intake				
Total cholesterol (mg)	489.31 ± 131.56	820.85 ± 162.36	−16.155	**<0.001**
Total energy(kcal)	1657.35 ± 741.41	1509.65 ± 650.60	−2.421	**0.015**
Saturated fatty acid (g)	14.00 ± 4.77	16.83 ± 3.52	−5.974	**<0.001**
monounsaturated fatty acid (g)	11.24 ± 3.95	12.45 ± 3.17	−3.690	**<0.001**
polyunsaturated fatty acid (g)	8.28 ± 5.59	7.83 ± 4.29	−0.310	0.756
Characteristics at the postnatal assessment				
Weight(g)	3216.54 ± 482.38	3220.55 ± 444.66	−0.686	0.493
Sex of the newborn			0.245	0.621
Boy	98 (56.0)	102(58.6)		
Girl	77(44.0)	72(41.4)		
Macrosomia			2.715	0.099
No	171(97.7)	164(94.3)		
Yes	4 (2.3)	10 (5.7)		
Large for gestational age			1.436	0.231
No	164 (93.7)	157 (90.2)		
Yes	11 (6.3)	17 (9.8)		
Small for gestational age			1.873	0.171
No	162 (92.6)	167 (96.0)		
Yes	13 (7.4)	7 (4.0)		
Premature newborn			0.826	0.363
No	160 (91.4)	154 (88.5)		
Yes	15 (8.6)	20 (11.5)		
Cesarean section			0.024	0.877
No	99 (56.6)	97 (55.7)		
Yes	76 (43.4)	77(44.3)		
OGTT 0 h			1.535	0.215
Normal	133 (76.0)	122 (70.1)		
Abnormal	42 (24.0)	52 (29.9)		
OGTT 1 h			0.931	0.335
Normal	67 (38.3)	58 (33.3)		
Abnormal	108 (61.7)	116 (66.7)		
OGTT 2 h			0.087	0.768
Normal	64 (36.6)	61 (35.1)		
Abnormal	111 (63.4)	113 (64.9)		
HbA1C in the second trimester of pregnancy			1.405	0.236
Normal	171 (97.7)	166 (95.4)		
Abnormal	4 (2.3)	8 (4.6)		

*t: the statistics of the Mann–Whitney U test.*

*χ2: the statistics of the chi-square test.*

^*^Fisher’s exact test.

Bold values indicate that the *p*-value is less than 0.05, indicating statistical significance.

OGTT, oral glucose tolerance test.

### 3.2 Cholesterol intake in women with GDM

The average cholesterol intake among 349 GDM women in the second trimester was 654.60 ± 222.07 mg/d. The total energy intake was 1583.71 ± 700.53 kcal/d, the saturated fatty acid intake was 15.41 ± 4.42 g/d, the monounsaturated fatty acid intake was 11.85 ± 3.63 g/d, and the polyunsaturated fatty acid intake was 8.06 ± 4.99 g/d. The average cholesterol intake of 175 GDM women in the low cholesterol intake group and 174 women in the high cholesterol intake group was 489.31 ± 131.56 mg/d and 820.85 ± 162.36 mg/d, respectively. There were also significant differences in energy, saturated fatty acid, and monounsaturated fatty acid intakes between the low and high cholesterol intake groups, while there was no significant difference in polyunsaturated fatty acid intakes between the two groups. The average daily energy intake of GDM women in the low and high cholesterol intake groups was 1657.35 ± 741.41 kcal and 1509.65 ± 650.60 kcal, respectively. All details are shown in Table 1.

Subsequent follow-up successfully obtained the dietary intake of 295 GDM women in the third trimester of pregnancy. Their cholesterol intake was 696.24 ± 563.07 mg/d, total energy intake was 1597.63 ± 563.07 kcal/d, saturated fatty acid intake was 15.67 ± 4.07 g/d, monounsaturated fatty acid intake was 11.36 ± 3.15 g/d, and polyunsaturated fatty acid intake was 5.79 ± 2.95 g/d. The average cholesterol intake of 148 GDM women in the low cholesterol intake group and 147 women in the high cholesterol intake group was 560.42 ± 95.17 mg/d and 832.98 ± 182.86 mg/d, respectively. There were also significant differences in energy, saturated fatty acids, monounsaturated fatty acids, and polyunsaturated fatty acids between the low and high cholesterol intake groups. There was no significant difference in polyunsaturated fatty acid intake between the two groups. The average daily energy intake of pregnant women in the low and high cholesterol intake groups was 1712.54 ± 624.49 kcal and 1481.94 ± 467.90 kcal, respectively. All details are shown in Supplementary Table S1.

**Figure 1:**
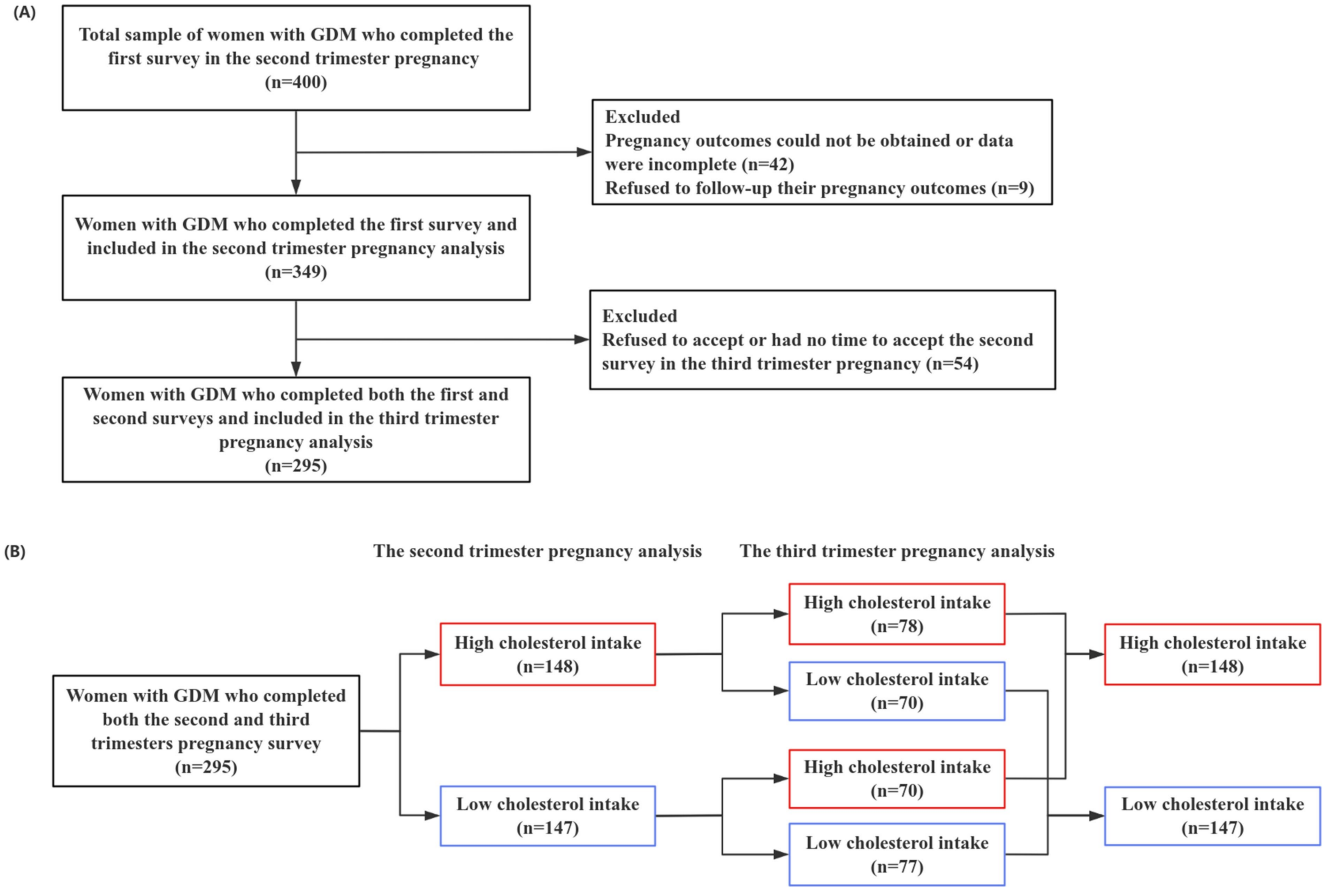
**(A)** Flow chart for the inclusion and exclusion of the study population. **(B)** Reflects the cholesterol intake grouping in the second and third trimesters of pregnancy of 295 women with GDM who completed both the second and third trimesters of the pregnancy survey.

Among the 295 GDM women who completed the second and third trimesters of pregnancy surveys, some of their cholesterol intake changed. On the one hand, 148 GDM women were in the high cholesterol intake group in the second trimester of pregnancy. However, in the third trimester of pregnancy, 78 were still in the high cholesterol intake group, while 70 were changed in the low cholesterol intake group. On the other hand, a total of 147 GDM women were in the low cholesterol intake group in the second trimester of pregnancy. Nevertheless, in the third trimester of pregnancy, 77 were still in the low-cholesterol intake group, while 70 were in the high-cholesterol intake group (Figure 1).

### 3.3 Blood glucose levels and pregnancy outcomes in GDM women

Table 1 and Supplementary Table S1 also show the blood glucose levels and pregnancy outcomes in GDM women during the second and third trimesters of pregnancy. In the second trimester of pregnancy, 26.93% of GDM women had abnormal fasting blood glucose, and 64.18% of women had abnormal blood glucose at 1 h and 2 h after drinking sugar water, and 3.44% of them had abnormal HbA1C (Table 1). In the third trimester of pregnancy, 10.85% of GDM women had abnormal fasting blood glucose, 26.44% of women had abnormal 2-h postprandial blood glucose, and 11.86% of women had abnormal HbA1C (Supplementary Table S1). Among the 349 GDM women analyzed in the second trimester, the average birth weight of newborns was 3218.54 ± 463.29 g, of which 4.01% were macrosomia, 8.02% were LGA, 5.73% were SGA, and 10.03% were premature newborns (Table 1). Among the 295 GDM women included in the third trimester of pregnancy, the average birth weight of newborns was 3215.22 ± 467.12 g. Macrosomia accounted for 4.41%, LGA accounted for 8.14%, SGA accounted for 5.76%, and premature newborns accounted for 10.17% (Supplementary Table S1). Overall, there was no significant difference in maternal blood glucose and pregnancy outcome in GDM women between the low and high cholesterol intake groups (*p* > 0.05).

### 3.4 The relationship between cholesterol intake and blood glucose in women with GDM

We explored the relationship between cholesterol intake and blood glucose in GDM women by logistic regression analyses. In the second trimester of pregnancy, the logistic regression model results showed that GDM women with low cholesterol intake had a higher risk of abnormal HbA1C compared with women with high cholesterol intake. The difference was statistically significant in the calibration model (OR = 26.014, 95% CI = 2.616–258.727, *p* < 0.01) (Table 2). However, the level of cholesterol intake during the second trimester of pregnancy was not associated with OGTT blood glucose values in GDM women (*p* > 0.05) (Table 2).

**Table 2:** The relationship between blood glucose and pregnancy outcome of GDM women and cholesterol intake in the second trimester of pregnancy.

Characteristic	Crude model		Adjusted model	
	OR(95%CI)^a^	*p*	OR(95%CI)	*p*
Pregnancy outcomes				
Macrosomia	2.607 (0.802, 8.476)	0.111	23.195 (2.650, 203.024)	**0.005** ^**b**^
Large for gestational age	1.614 (0.733, 3.555)	0.234	3.253 (1.062, 9.965)	**0.039** ^**b**^
Small for gestational age	0.522 (0.203, 1.342)	0.178	0.271 (0.074, 0.986)	**0.047** ^**b**^
Cesarean section	1.034 (0.677, 1.578)	0.877	1.438 (0.810, 2.553)	0.215^c^
Blood glucose situation				
OGTT 0 h	1.350 (0.839, 2.171)	0.216	1.361 (0.741, 2.501)	0.320^d^
OGTT 1 h	1.241 (0.800, 1.924)	0.335	1.399 (0.797, 2.454)	0.242^d^
OGTT 2 h	1.068 (0.689, 1.655)	0.768	0.965 (0.548, 1.698)	0.901^d^
HbA1C in the second trimester of pregnancy	2.060 (0.609, 6.972)	0.245	26.014 (2.616, 258.727)	**0.005** ^**d**^

a Odds ratio and confidence intervals (95% CI).

b Adjusted Model: adjusted for age, pre-pregnancy body mass index, number of pregnancies, whether or not the woman is primiparous, education level, whether to work during pregnancy, family per capita monthly income, hypertension before pregnancy, abnormal lipid metabolism before pregnancy, exercise frequency during the second trimester of pregnancy, extra meals during the second trimester of pregnancy, total energy intake, protein intake, carbohydrate intake, saturated fatty acid intake, monounsaturated fatty acid intake, and polyunsaturated fatty acid intake.

c Adjusted Model: adjusted for age, pre-pregnancy body mass index, number of pregnancies, whether or not the woman is primiparous, education level, whether to work during pregnancy, family per capita monthly income, hypertension before pregnancy, abnormal lipid metabolism before pregnancy, exercise frequency during the second trimester of pregnancy, extra meals during the second trimester of pregnancy, total energy intake, protein intake, carbohydrate intake, saturated fatty acid intake, monounsaturated fatty acid intake, and polyunsaturated fatty acid intake.

d Adjusted Model: adjusted for age, pre-pregnancy body mass index, number of pregnancies, whether or not the woman is primiparous, education level, whether to work during pregnancy, family per capita monthly income, family history of diabetes, exercise frequency during the second trimester of pregnancy, extra meals during the second trimester of pregnancy, total energy intake, protein intake, carbohydrate intake, saturated fatty acid intake, monounsaturated fatty acid intake, and polyunsaturated fatty acid intake.

Bold values indicate that the *p*-value is less than 0.05, indicating statistical significance.

In the third trimester of pregnancy, the regression results suggested that compared with GDM women with high cholesterol intake, GDM women with low cholesterol intake had higher risks of abnormal HbA1C and abnormal venous fasting blood glucose, and these differences were statistically significant in the correction model (OR = 2.773, 95% CI = 1.028–7.482, *p* < 0.05; and OR = 2.907, 95% CI = 1.011–8.360, *p* < 0.05, respectively) (Table 3). However, the level of cholesterol intake in the third trimester of pregnancy was not associated with 2-h postprandial blood glucose in GDM women (*p* > 0.05) (Table 3). The adjusted model for the third trimester of pregnancy not only included all the covariates in the second trimester of pregnancy adjusted model but also increased the number of nutritional outpatient interventions for GDM women during pregnancy.

**Table 3:** The relationship between blood glucose, pregnancy outcome, and cholesterol intake in the third trimester of pregnancy in GDM women.

Characteristic	Crude model		Adjusted model	
	OR(95%CI)^a^	*p*	OR(95%CI)	*p*
Pregnancy outcomes				
Macrosomia	0.616 (0.197, 1.930)	0.406	0.023 (0.001, 0.436)	**0.012** ^**b**^
Large for gestational age	0.578 (0.245, 1.367)	0.212	0.199 (0.042, 0.949)	**0.043** ^**b**^
Small for gestational age	1.141 (0.428, 3.044)	0.792	0.417 (0.062, 2.807)	0.369^b^
Cesarean section	1.373 (0.864, 2.180)	0.179	1.987 (1.008, 3.916)	**0.047** ^**c**^
Blood glucose situation				
Venous fasting blood glucose in the third trimester of pregnancy	1.758 (0.826, 3.742)	0.143	2.907 (1.011, 8.360)	**0.048** ^**d**^
Blood glucose 2 h after intravenous meal in the third trimester of pregnancy	1.310 (0.779, 2.203)	0.308	1.277 (0.637, 2.560)	0.491^d^
HbA1C in the third trimester	1.800 (0.869, 3.725)	0.113	2.773 (1.028, 7.482)	**0.044** ^**d**^

a Odds ratio and confidence intervals (95% CI).

b Adjusted Model: adjusted for age, pre-pregnancy body mass index, whether or not the woman is primiparous, education level, whether to work during pregnancy, family per capita monthly income, couples living together, family history of obesity, hypertension before pregnancy, abnormal lipid metabolism before pregnancy, thalassemia before pregnancy, exercise frequency during the third trimester of pregnancy, extra meals during the third trimester of pregnancy, eating habits in the third trimester of pregnancy, OGTT results, total energy intake, protein intake, carbohydrate intake, saturated fatty acid intake, monounsaturated fatty acid intake, and polyunsaturated fatty acid intake.

c Adjusted Model: adjusted for age, pre-pregnancy body mass index, number of pregnancies, whether or not the woman is primiparous, education level, whether to work during pregnancy, family per capita monthly income, couples living together, family history of obesity, family history of diabetes, thalassemia before pregnancy, exercise frequency during the third trimester of pregnancy, extra meals during the third trimester of pregnancy, number of nutrition clinic interventions, fetal age, OGTT results, total energy intake, protein intake, carbohydrate intake, saturated fatty acid intake, monounsaturated fatty acid intake, and polyunsaturated fatty acid intake.

d Adjusted Model: adjusted for age, smoking history, number of pregnancies, education level, whether to work during pregnancy, family per capita monthly income, couples living together, family history of diabetes, GDM before pregnancy, thalassemia before pregnancy, exercise frequency during the third trimester of pregnancy, extra meals during the third trimester of pregnancy, number of nutrition clinic interventions, total energy intake, protein intake, carbohydrate intake, saturated fatty acid intake, monounsaturated fatty acid intake, and polyunsaturated fatty acid intake.

Bold values indicate that the *p*-value is less than 0.05, indicating statistical significance.

### 3.5 The relationship between cholesterol intake and pregnancy outcome in women with GDM

We also explored the relationship between cholesterol intake and the pregnancy outcome of GDM women via logistic regression analyses. In the second trimester of pregnancy, the regression results indicated that GDM women with high cholesterol intake had higher risks of macrosomia and LGA than those with low cholesterol intake, and these differences were statistically significant in the calibration model (OR = 23.195, 95% CI = 2.650–203.024, *p* < 0.01; and OR = 3.253, 95% CI = 1.062–9.965, *p* < 0.05, respectively) (Table 2). In addition, high cholesterol intake in GDM women also reduced the risk of SGA, and this difference was still statistically significant in the calibration model (OR = 0.271, 95% CI = 0.074–0.986, *p* < 0.05) (Table 2).

However, in the third trimester of pregnancy, the regression results showed that GDM women with high cholesterol intake had lower risks of macrosomia and LGA than those with low cholesterol intake, and this difference was statistically significant in the correction model (OR = 0.023, 95% CI = 0.001–0.436, *p* < 0.05; and OR = 0.199, 95% CI = 0.042–0.949, *p* < 0.05, respectively) (Table 3).

In addition, logistic regression analysis showed that the cholesterol intake of women with GDM in the second trimester of pregnancy had no effect on the mode of delivery (*p* > 0.05) (Table 2), while high cholesterol intake in the third trimester of pregnancy increased the risk of cesarean section in the correction model (OR = 1.987, 95% CI = 1.008–3.916, *p* < 0.05) (Table 3).

## 4 Discussion

In this prospective cohort study, we found that GDM women generally had high cholesterol intake during pregnancy, and cholesterol intake in the third trimester of pregnancy was higher than in the second trimester. Low cholesterol intake in GDM women during the second and third trimesters of pregnancy increased the risk of abnormal HbA1C levels during the same period. The level of cholesterol intake in the second trimester of pregnancy was not related to the OGTT results, while the low cholesterol intake in the third trimester increased the risk of abnormal fasting blood glucose. Additionally, compared with GDM women with low cholesterol intake, GDM women with high cholesterol intake in the second trimester of pregnancy increased the risks of macrosomia and LGA, while GDM women with high cholesterol intake in the third trimester of pregnancy decreased their risk of macrosomia and LGA. In addition, high cholesterol intake during the second trimester of pregnancy also reduced the risk of SGA, and high cholesterol intake during the third trimester of pregnancy increased the risk of cesarean section.

Dietary factors play an important role in the occurrence of GDM, and higher cholesterol intake can promote the risk of developing GDM (34). A prospective cohort study in China showed that high total dietary cholesterol intake during pregnancy increased fasting blood glucose and 1-h postprandial blood glucose in the second trimester, and egg-derived cholesterol intake increased fasting blood glucose (22). This suggests that dietary cholesterol, especially egg-derived cholesterol intake, has a significant impact on blood glucose. The results of this study indicated that low cholesterol intake in the third trimester of pregnancy could increase the risk of abnormal venous fasting blood glucose in GDM women. This may be because the main source of cholesterol in GDM women participating in this study is eggs, and compared with pregnant women in the high cholesterol intake group, GDM women in the low cholesterol intake group have higher egg-derived cholesterol intake and its proportion of total cholesterol intake, so low total cholesterol intake might instead increase the risk of abnormal fasting blood glucose.

Moreover, HbA1C is the gold standard for reflecting the average level of long-term blood glucose control and is a recognized indicator of blood glucose levels over the past 2–3 months (35). Therefore, this study also explored the effect of cholesterol intake during pregnancy on HbA1C in GDM women. We found low cholesterol intake in GDM women during the second and third trimesters of pregnancy would increase the risk of abnormal HbA1C levels during the same period. Cholesterol intake is only one of the influencing factors of HbA1c, not its determinants. The average blood glucose level is one of the main determinants of HbA1c. Insulin resistance and *β*-cell damage are key components of the pathophysiology of GDM (36). The animal study has shown that the number of β cells is an important determinant of glucose homeostasis (37). And β-cell dysfunction is exacerbated by insulin resistance. HbA1c is one of the main indicators of average blood glucose level. Therefore, HbA1c is also indirectly affected by insulin resistance and β-cell damage. In addition, some of the participants with abnormal HbA1c in the second trimester of pregnancy of this study belonged to the high cholesterol intake group in the second trimester of pregnancy, while were assigned to the low cholesterol intake group in the third trimester of pregnancy. Although the cholesterol intake of these pregnant women is relatively reduced in the third trimester of pregnancy, they may have severe damage to β cells in the second trimester of pregnancy, so after dietary intervention, insulin was still insufficiently secreted. The average blood glucose level was higher, which made the HbA1c level of them still higher in the third trimester of pregnancy. Therefore, GDM women with lower cholesterol intake might have higher risks of abnormal HbA1c levels.

Our results suggested that high cholesterol intake in the second trimester of pregnancy increased the risks of LGA and macrosomia in women with GDM. A study found that the dietary cholesterol intake of low-income women during pregnancy affected fetal growth, and those with high cholesterol intake (195.8 mg/1,000 kcal) were more likely to give birth to LGA babies than women with low cholesterol intake (148 mg/1,000 kcal) (21). Women with cholesterol intake in the fourth quartile (183.5–466.7 mg/1,000 kcal) were 2.48 times more at risk of giving to LGA newborns than women in the first to third quartiles (48.0–182.6 mg/1,000 kcal) (95% CI = 1.31–4.66) (21). This is consistent with the results of the second trimester of pregnancy in this study. High dietary cholesterol intake may increase serum cholesterol, affecting blood glucose and contributing to adverse pregnancy outcomes such as macrosomia.

Regarding the relationship between dietary cholesterol intake and serum cholesterol levels, previous metabolic studies have shown that dietary cholesterol might increase serum cholesterol concentrations (15, 16). Kaneko et al. also showed that maternal serum total cholesterol increased by 1-SD and was linearly correlated with LGA (OR = 1.13; 95% CI = 1.09–1.16). In contrast, maternal serum cholesterol decreased 1-SD was linearly correlated with SGA (OR = 1.20; 95% CI = 1.15–1.25) (38). We also found that compared with GDM women with low cholesterol intake (560.42 ± 95.17 mg/d), GDM women with high cholesterol intake (832.98 ± 182.86 mg/d) reduced the risk of SGA. Xue et al. also found that maternal total cholesterol intake was negatively correlated with the risk of SGA, and the risk of SGA was significantly increased in women with the lowest cholesterol intake, which was consistent with the results of this study (33). Studies have shown that maternal cholesterol levels could determine the cholesterol levels of offspring, and maternal cholesterol plays an important role in placental synthesis (39). Evidence from mouse models shows that an increase in maternal serum total cholesterol concentration might lead to an increase in placental size, and a high placental surface area might lead to an increase in placental nutrient transfer capacity, thereby affecting fetal growth (40). Therefore, pregnant women with high cholesterol intake were more likely to provide adequate nutrition for the fetus so that the growth and organ development of the fetus are perfect, and the incidence of SGA was also reduced.

In this study, maternal cholesterol intake during the second trimester of pregnancy had an effect on both LGA and SGA. This might be because cholesterol intake is only one of the factors affecting LGA and SGA, and they were also affected by factors such as pre-pregnancy BMI. Studies have shown that SGA was independently associated with pre-pregnancy overweight or obesity, and overweight or obesity was associated with increased risk of LGA and decreased risk of SGA (41). This study showed a statistically significant difference in pre-pregnancy BMI between the two groups of pregnant women. Among them, 37.9% of participants in the high cholesterol intake group were overweight or obese, while only 22.9% of participants in the low cholesterol intake group were overweight or obese. Therefore, under the influence of pre-pregnancy BMI, high cholesterol intake may have an impact on both LGA and SGA.

Energy and protein intake were the most important maternal dietary factors affecting birth weight (42, 43). Previous studies have shown that total energy intake during pregnancy was positively correlated with neonatal birth weight (43–46). According to the “Dietary Guidelines for Patients with Gestational Diabetes Mellitus,” the daily extra calorie intake of GDM women in the first, second, and third trimesters of pregnancy in China is 300 kcal and 450 kcal, respectively (47). In this study, the energy intake of GDM women in the low cholesterol intake group in the second trimester of pregnancy (1657.35 ± 741.41 kcal/d) and the third trimester of pregnancy (1712.54 ± 624.49 kcal/d) was higher than that of GDM women in the high cholesterol intake group (the second trimester of pregnancy was 1509.65 ± 650.60 kcal/d, and the third trimester of pregnancy was 1481.94 ± 467.90 kcal/d). Meanwhile, the difference in energy intake between the two groups of GDM women in the third trimester of pregnancy was more obvious, and it was significantly lower than the recommended energy intake for GDM women recommended by the guidelines. In addition, the cholesterol intake of the two groups in the third trimester of pregnancy (low cholesterol intake group: 560.42 ± 95.17 g/d, high cholesterol intake group: 832.98 ± 182.86 g/d) was higher than that in the second trimester of pregnancy (low cholesterol intake group: 489.31 ± 131.56 g/d, high cholesterol intake group: 820.85 ± 162.36 g/d). However, GDM women with lower cholesterol intake in the third trimester of pregnancy had higher energy intake, so the intake of other dietary components, such as protein, might be higher in these women. In addition, participants in this study varied greatly in the grouping of high-cholesterol and low-cholesterol diets during the second and third trimesters of pregnancy by nearly 50%. This may be because, in the context of traditional Chinese culture, women with GDM still have a high level of cholesterol intake even after the intervention of clinicians and the change of diet in the third trimester of pregnancy. Therefore, GDM women with high cholesterol intake in the third trimester of pregnancy might have reduced risks of macrosomia and LGA compared with women with low cholesterol intake.

This study also found that high cholesterol intake in the third trimester of pregnancy of women with GDM would increase the incidence of cesarean section. A meta-analysis of the effects of dietary methods and exercise interventions on GDM found that the Dietary Approaches to Stop Hypertension (DASH) diet could reduce the rate of cesarean section by 46 % (48). The DASH diet is a healthy diet rich in fruits, vegetables, low-fat dairy products and whole grains, while limiting the intake of saturated fat, cholesterol, refined grains, refined sugar and sodium (49).

It is necessary to acknowledge the limitations of this work. First, participants self-reported their dietary cholesterol intake and assessed it by FFQ. Although all investigators were trained on how to use FFQ and used a consistent investigation order and inquiry methods in the investigation process to ensure the reliability and consistency of the data as much as possible, the results of the survey might still be affected by recall bias and measurement bias, so we could not rule out the possibility of underestimating or overestimating dietary intake. Secondly, this study lacked relevant information on serum cholesterol concentration or other blood lipid indicators in GDM women. Third, the questionnaires on physical activity and energy expenditure were not used; however, we only used the frequency of exercise during pregnancy, which might make the assessment of physical activity and energy expenditure less accurate. Finally, like other observational studies, we still could not rule out the possibility of unobserved confounders even after controlling for many potential confounders, including sociodemographic, dietary, disease, and family history factors.

## 5 Conclusion

Overall, the results of this study showed that compared with GDM women with high cholesterol intake, GDM women with low cholesterol intake during the second and third trimesters of pregnancy had higher risks of abnormal HbA1C during the same period. GDM women with low cholesterol intake also had higher risks of abnormal fasting blood glucose in the third trimester of pregnancy. Meanwhile, in the second trimester of pregnancy, GDM women with high cholesterol intake had higher risks of macrosomia and LGA than those with low cholesterol intake. However, in the third trimester of pregnancy, GDM women with high cholesterol intake had lower risks of macrosomia and LGA than those with low cholesterol intake. GDM women with high cholesterol intake during the second trimester of pregnancy also had lower risks of SGA than those with low cholesterol intake. In addition, high cholesterol intake during the third trimester of pregnancy increased the risk of cesarean section. Dietary cholesterol intake during pregnancy influences blood glucose control in GDM women and may increase the risk of adverse pregnancy outcomes, which has a negative impact on maternal and child health. Therefore, cholesterol intake in GDM women should be limited to reasonable levels for better blood glucose control and to reduce the incidence of adverse pregnancy outcomes. In the future, we need to collect lipid-related data of GDM women for more comprehensive research to further analyze the mechanism of the relationship between dietary cholesterol and blood glucose and pregnancy outcomes.

## Data Availability

The raw data supporting the conclusions of this article will be made available by the authors, without undue reservation.
