# Supplementary material for: Association of maternal dietary cholesterol intake during the second and third trimesters of pregnancy and blood glucose and pregnancy outcome in women with gestational diabetes mellitus: a prospective cohort study
Source: Front Nutr. 2024 Dec 12;11:1449000. doi: 10.3389/fnut.2024.1449000 (PMC11670664; doi:10.3389/fnut.2024.1449000)
Supplement: Supplementary file 1 [file Table_1.DOCX]

**Table S1. The characteristics of 295 GDM women were analyzed according to the level of cholesterol intake in the third trimester pregnancy.**

| **Characteristics** | **Total dairy products** | | ***Z*/χ^2^** | ***P*** |
| --- | --- | --- | --- | --- |
|  | **Less intake**  **n=148** | **More intake**  **n=147** |  |  |
| Baseline characteristics | | | | |
| Age | 31.30±3.86 | 32.41±3.90 | -2.158 | **0.031** |
| Nation |  |  | 1.362 | 0.243 |
| Han nationality | 140(94.6) | 143(97.3) |  |  |
| Minority | 8(5.4) | 4(2.7) |  |  |
| Education level |  |  | 2.716 | 0.438 |
| Junior high school and below | 19(12.8) | 11(7.5) |  |  |
| High school or technical secondary school | 17(11.5) | 16(10.9) |  |  |
| College for professional training | 42(28.4) | 41(27.9) |  |  |
| Bachelor degree and above | 70(47.3) | 79(53.7) |  |  |
| Work during pregnancy |  |  | 0.028 | 0.867 |
| No | 111(75.0) | 109(74.1) |  |  |
| Yes | 37(25.0) | 38(25.9) |  |  |
| Family per capita monthly income |  |  | 5.770 | 0.056 |
| ＜6000yuan | 52(35.1) | 39(26.5) |  |  |
| 6000~8999yuan | 60(40.5) | 54(36.7) |  |  |
| ≥9000yuan | 36(24.3) | 54(36.7) |  |  |
| Couples live together |  |  | 2.916 | 0.088 |
| No | 121(81.8) | 108(73.5) |  |  |
| Yes | 27(18.2) | 39(26.5) |  |  |
| Body mass index (kg/m^2^) |  |  | 0.636 | 0.728 |
| ＜18.5 | 15(10.1) | 14(9.5) |  |  |
| 18.5-23.9 | 91(61.5) | 85(57.8) |  |  |
| ≥24 | 42(28.4) | 48(32.7) |  |  |
| Number of pregnancies |  |  | 0.293 | 0.588 |
| First pregnancy | 64(43.2) | 59(40.1) |  |  |
| Multiple pregnancies | 84(56.8) | 88(59.9) |  |  |
| Primiparous woman |  |  | 1.257 | 0.262 |
| Yes | 95(64.2) | 85(57.8) |  |  |
| No | 53(35.8) | 62(42.2) |  |  |
| Cesarean section experience |  |  | 3.262 | 0.071 |
| No | 131(88.5) | 119(81.0) |  |  |
| Yes | 17(11.5) | 28(19.0) |  |  |
| Smoking history |  |  | 0.000 | 1.000 |
| No | 147(99.3) | 146(99.3) |  |  |
| Yes | 1(0.7) | 1(0.7) |  |  |
| Family history of obesity |  |  | 9.096 | **0.003** |
| No | 140(94.6) | 123(83.7) |  |  |
| Yes | 8(5.4) | 24(16.3) |  |  |
| Family history of diabetes |  |  | 5.995 | **0.014** |
| No | 113(76.4) | 93(63.3) |  |  |
| Yes | 35(23.6) | 54(36.7) |  |  |
| Family history of hypertension |  |  | 2.583 | 0.108 |
| No | 114(77.0) | 101(68.7) |  |  |
| Yes | 34(23.0) | 46(31.3) |  |  |
| Pre-pregnancy history of gestational diabetes mellitus |  |  | 0.049 | 0.826 |
| No | 135(91.2) | 133(90.5) |  |  |
| Yes | 13(8.8) | 14(9.5) |  |  |
| History of pre-pregnancy hypertension |  |  | 1.361 | 0.243 |
| No | 148(100.0) | 144(98.0) |  |  |
| Yes | 0(0.0) | 3(2.0) |  |  |
| Abnormal liver and kidney function before pregnancy |  |  | 0.000 | 1.000 |
| No | 145(98.0) | 145(98.6) |  |  |
| Yes | 3(2.0) | 2(1.4) |  |  |
| Abnormal lipid metabolism before pregnancy |  |  | 0.260 | 0.610 |
| No | 147(99.3) | 144(98.0) |  |  |
| Yes | 1(0.7) | 3(2.0) |  |  |
| Pre-pregnancy presence of thalassemia |  |  | 6.569 | **0.010** |
| No | 139(93.9) | 146(99.3) |  |  |
| Yes | 9(6.1) | 1(0.7) |  |  |
| Patients with thyroid metabolic diseases |  |  | 0.064 | 0.801 |
| No | 139(93.9) | 137(93.2) |  |  |
| Yes | 9(6.1) | 10(6.8) |  |  |
| Exercise frequency in the third trimester pregnancy |  |  | 0.277 | 0.964 |
| None | 19(12.8) | 20(13.6) |  |  |
| 1~2 days / week | 48(32.4) | 51(34.7) |  |  |
| 3~5 days / week | 55(37.2) | 52(35.4) |  |  |
| 6~7 days / week | 26(17.6) | 24(16.3) |  |  |
| The average sleep time in the past 3 months |  |  | 0.010 | 0.922 |
| ＜8 hours | 109(73.6) | 109(74.1) |  |  |
| ≥8 hours | 39(26.4) | 38(25.9) |  |  |
| Extra meals during the third trimester pregnancy |  |  | 1.151 | 0.765 |
| None | 3(2.0) | 4(2.7) |  |  |
| 1~2 days / week | 30(20.3) | 31(21.1) |  |  |
| 3~5 days / week | 46(31.1) | 52(35.4) |  |  |
| 6~7 days / week | 69(46.6) | 60(40.8) |  |  |
| Eating habits in the third trimester pregnancy |  |  | 0.254 | 0.881 |
| slack | 87(58.8) | 85(57.8) |  |  |
| Normal | 58(39.2) | 60(40.8) |  |  |
| Rich | 3(2.0) | 2(1.4) |  |  |
| Number of nutrition clinic interventions |  |  | 0.408 | 0.523 |
| ≤2 | 80(54.1) | 74(50.3) |  |  |
| ≥3 | 68(45.9) | 73(49.7) |  |  |
| Daily intake |  |  |  |  |
| Total cholesterol (mg) | 560.42±95.17 | 832.98±182.86 | -14.849 | **＜0.001** |
| Egg-derived cholesterol (mg) | 437.61±208.57 | 425.91±214.82 | -0.072 | 0.943 |
| Total energy（kcal） | 1712.54±624.49 | 1481.94±467.90 | -2.800 | **0.005** |
| Saturated fatty acid (g) | 14.15±3.77 | 17.20±3.79 | -6.309 | **＜0.001** |
| Monounsaturated fatty acid (g) | 10.27±2.83 | 12.46±3.07 | -6.024 | **＜0.001** |
| Polyunsaturated fatty acid (g) | 5.56±3.22 | 6.03±2.63 | -2.722 | **0.006** |
| Characteristics at the postnatal assessment |  |  |  |  |
| Weight（g） | 3232.43±484.01 | 3197.89±450.46 | -0.246 | 0.806 |
| Sex of the newborn |  |  | 0.028 | 0.866 |
| Boy | 84(56.8) | 82(55.8) |  |  |
| Girl | 64(43.2) | 65(44.2) |  |  |
| Macrosomia |  |  | 0.703 | 0.402 |
| No | 140(94.6) | 142(96.6) |  |  |
| Yes | 8(5.4) | 5(3.4) |  |  |
| Large for gestational age |  |  | 1.589 | 0.207 |
| No | 133(89.9) | 138(93.9) |  |  |
| Yes | 15(10.1) | 9(6.1) |  |  |
| Small for gestational age |  |  | 0.070 | 0.792 |
| No | 140(94.6) | 138(93.9) |  |  |
| Yes | 8(5.4) | 9(6.1) |  |  |
| Premature newborn |  |  | 0.564 | 0.453 |
| No | 131(88.5) | 134(91.2) |  |  |
| Yes | 17(11.5) | 13(8.8) |  |  |
| Cesarean section |  |  | 1.807 | 0.179 |
| No | 90(53.6) | 58(45.7) |  |  |
| Yes | 78(46.4) | 69(54.3) |  |  |
| OGTT 0h |  |  | 0.462 | 0.497 |
| Normal | 108(73.0) | 102(69.4) |  |  |
| Abnormal | 40(27.0) | 45(30.6) |  |  |
| OGTT 1h |  |  | 0.795 | 0.373 |
| Normal | 50(33.8)  98(66.2) | 57(38.8) |  |  |
| Abnormal |  | 90(61.2) |  |  |
| OGTT 2h |  |  | 1.675 | 0.196 |
| Normal | 58(39.2) | 47(32.0) |  |  |
| Abnormal | 90(60.8) | 100(68.0) |  |  |
| Venous fasting blood glucose in the third trimester pregnancy |  |  | 2.183 | 0.140 |
| Normal | 128(86.5) | 135(91.8) |  |  |
| Abnormal | 20(13.5) | 12(8.2) |  |  |
| Blood glucose 2 hours after intravenous meal in the third trimester pregnancy |  |  | 1.043 | 0.307 |
| Normal | 105(70.9) | 112(76.2) |  |  |
| Abnormal | 43(29.1) | 35(23.8) |  |  |
| HbA1C in the third trimester pregnancy |  |  | 2.557 | 0.110 |
| Normal | 126(85.1) | 134(91.2) |  |  |
| Abnormal | 22(14.9) | 13(8.8) |  |  |

*t*：the statistics of Mann-Whitney U test.

χ^2^：the statistics of chi-square test.

Bold values indicates that the *p* value is less than 0.05, indicating statistical significance.

OGTT: oral glucose tolerance test.
